# “Shelter-in-Place” Policies and Changes in Caregiving for Older Adults During the COVID-19 Pandemic

**DOI:** 10.3390/ijerph23070825

**Published:** 2026-06-23

**Authors:** Lei Chen, Joanne Spetz

**Affiliations:** Philip R. Lee Institute for Health Policy Studies, University of California, San Francisco, CA 94158, USA; joanne.spetz@ucsf.edu

**Keywords:** “Shelter-in-Place” policies, changes in caregiving, COVID-19 pandemic, unmarried older adults, people with disabilities

## Abstract

**Highlights:**

**Public health relevance—How does this work relate to a public health issue?**

- The COVID-19 pandemic and associated “Shelter-in-Place” policies disrupted caregiving arrangements for older adults, particularly those without spousal support—a group highly vulnerable to both infection and unmet care needs during public health emergencies.

**Public health significance—Why is this work of significance to public health?**

- Using nationally representative data, this study provides population-level evidence on how emergency policies may have unintended consequences for caregiving for older adults with functional impairments, particularly those who are not married.

**Public health implications—What are the key implications or messages for practitioners, policy makers and/or researchers in public health?**

- Public health and policy leaders should ensure that caregiving needs for people who have functional impairments and do not have spousal support are addressed during public health emergencies.

**Abstract:**

During the COVID-19 pandemic, family support for older adults living with disabilities was disrupted due to “Shelter-in-Place (SIP)” orders. This study examined the impact of SIP policies on caregiving changes for people with disabilities who were not married. We used the National Health and Aging Trends Survey (NHATS) round 10 data and previously published data regarding SIP policies. The study sample included NHATS community-dwelling respondents who needed assistance with activities of daily living (ADLs) or instrumental activities of daily living (IADLs) and who were not married (n = 512). More than half of people (55.1%) reported no change in receiving help, approximately one-third (36.7%) reported receiving less help, and 8.2% reported receiving more help during COVID-19 than before. Compared with people who lived in areas that had fewer than 30%, people living in areas with 30–59% and with 60% or more of SIP days had 12 percentage points lower probability of reporting they received more help during COVID-19 (*p* = 0.02 for 30–59% and *p* = 0.03 for ≥60%). It is crucial to address caregiving needs during public health emergencies and to examine how policy disruptions impact support for individuals reliant on family assistance.

## 1. Introduction

The number of Americans ages 65 and older is projected to increase from 58 million in 2022 to 82 million by 2050, a 42% increase, and this group of older adults’ share of the total population is projected to rise from 17% to 23% [1]. Growth in the population of older adults will contribute to rising demand for assistance with activities of daily living (ADLs; e.g., eating, bathing, dressing, toileting, and transferring) and instrumental activities of daily living (IADLs; i.e., higher-order activities like medication management and food preparation that allow a person to live independently) [2]. The need for support in daily living is compounded for the estimated 7.2 million people living with Alzheimer’s disease and related dementia [3]. Family members—particularly spouses—provide much of the assistance that older adults with functional needs require, and, thus, older adults who do not live with a spouse are at particular risk of not receiving sufficient support for their ADL and IADL needs [4,5].

Caregiving processes are shaped not only by individual factors, such as family support, but also by broader policy and structural contexts [6]. The vulnerabilities of older adults living with disabilities who do not live with a spouse were intensified during the COVID-19 pandemic, as “Shelter-in-Place (SIP)” orders reduced access to both formal services and informal caregiving networks due to mobility restrictions. “Shelter-in-place” refers to government-issued public health directives during the COVID-19 pandemic that required individuals to remain at home except for essential activities (e.g., obtaining food or medical care), commonly referred to as “lockdown” measures in some countries. On 13 March 2020, the U.S. White House declared a national emergency due to COVID-19. Soon after, many states and counties issued SIP orders to slow disease transmission. The timing, severity, and enforcement of state orders varied widely [7], and survey research has reported that people with disabilities faced disruptions due to discontinuation of supportive services, including transportation services, therapy, community cleaning and laundry services, and housing and employment programs [8]. There were also changes in living arrangements of adults with disabilities during the pandemic: the proportion of community-dwelling homebound adults aged 70 years or older substantially increased in the U.S. in 2020 [9]. Some individuals may have moved from residential care to family members’ homes, while others may have delayed transitioning to residential care. In addition, many adult children moved back in with their parents [10,11].

Most previous studies in the U.S. have examined the impact of COVID-19 on caregivers’ well-being, mental health, physical health, financial stability, and job outcomes by conducting systematic reviews or using national-level surveys, such as the National Health and Aging Trends Study (NHATS) and Health and Retirement Study [12,13,14,15]. Other online surveys and qualitative studies were conducted to explore family caregivers’ experiences, burden, caregiving intensity, and changes to family caregiving of older adults and adults with disabilities during COVID-19 [16,17,18]. Regarding adults with disabilities, one small survey conducted early in the pandemic (n = 109) reported that reductions in community services increased the need for adults with disabilities to rely on family members to provide personal care, but it is not known how widespread this might have been, as adults with disabilities faced discontinuation of services upon which they relied to meet their medical, physical, and social needs [8]. Previous studies also found that SIP policies during COVID-19 had a negative impact on older adults’ mental health and social well-being, generating higher rates of depression and greater loneliness [19]. However, less is known about how SIP policies affected changes in caregiving for older adults with daily living needs, particularly those who were not married and thus could not rely on spousal support.

This study used nationally representative survey data from the U.S. to examine patterns of help provided during the COVID-19 pandemic for people aged 65 years and older who needed assistance with daily living activities and did not have spousal support. By describing the survey’s findings, we offer new insights into caregiving for unmarried older adults who need assistance with daily living activities during public health emergencies. We also contribute to the literature by investigating changes in caregiving by dementia status and ADL/IADL needs, as well as the impact of SIP policies on changes in caregiving during public health emergencies such as the COVID-19 pandemic. We hypothesized that greater SIP policy intensity is associated with a higher likelihood of reduced caregiving among older adults.

## 2. Materials and Methods

### 2.1. Study Design and Data

This study is a nationally representative, observational study using cross-sectional data from the NHATS Round 10 (fielded from May 2020 to October 2020) and the NHATS COVID-19 supplemental survey (fielded from June 2020 to March 2021), with weighted analyses to account for the complex survey design. The NHATS data include survey data for respondents and a separate data file that provides data from surveys conducted of family and friends identified by the respondent as providing support. If an NHATS respondent was unable to complete the survey themselves, another individual could assist in completing the survey (“proxy respondent”). The COVID-19 module was administered to NHATS participants who completed the Round 10 interview (including proxy respondents) and thus represents a subset of the full NHATS panel. The COVID-19 files include detailed information on participants’ experiences during the pandemic, including changes in daily activities, social contact, and caregiving received. For this study, we used the restricted-use geographic data files, which provide geographic information about respondents’ residential location (state, county, and metropolitan area codes). The geographic indicators are available for both the NHATS Round 10 and the COVID-19 supplemental survey respondents [20,21]. We linked the NHATS COVID-19 geographic data files with published data regarding SIP policies at the state and county levels [7]. Because all data were secondary, the Institutional Review Board determined that the study was eligible for expedited review under Category 5 and certified its approval of the Restricted Data Protection Plan created for the study.

### 2.2. Participants

**Figure 1:**
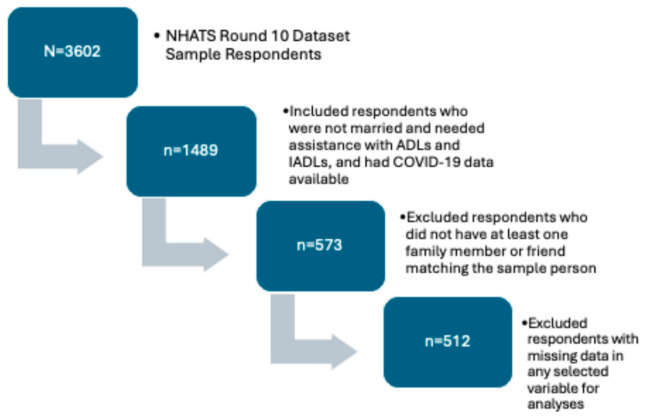
NHATS Round 10 Analytic Respondents Sample.

We began with 3602 community-dwelling respondents who completed the Round 10 survey. This sample was then limited to 1489 respondents who were not married, needed assistance with ADLs (eating, bathing, toileting, and dressing) or IADLs (laundry, shopping, meal preparation, paying bills, or tracking prescription medication), and had COVID-19 data available. We then merged the data between the sample person and family members and friends files, because the question on changes in caregiving was included in these files. We identified 573 unique sample persons who had at least one family member or friend matching the sample person. The final analytic data file consisted of 512 unique complete-case respondents with no missing data for all measurements. Figure 1 shows a flow diagram illustrating this sample selection process.

### 2.3. Measurements

#### 2.3.1. Outcome

We used the measurement of changes in caregiving received by family members and friends of the sample person before and during COVID-19 as the outcome from the NHATS restricted COVID-19 Files. The questionnaire asked, “During the COVID-19 outbreak, have you helped the NHATS participant more, less, or about the same compared to a typical week before the outbreak? [12]” This item was coded as a categorical variable with three levels: no change in help during COVID-19, less help during COVID-19, and more help during COVID-19.

#### 2.3.2. Predictor

The measure of SIP policies was based on data on the percentage of days between 24 February and 30 May 2020, that county residents were under a SIP order [7]. Most of the variation in SIP orders came from state policies, although some counties implemented their own orders before their states did so; thus, we used data at the county level. SIP orders included “stay-at-home”, “safer-at-home,” and related orders, as well as policies ordering the closure of nonessential businesses [7]. This measure was originally specified as a continuous variable measuring the percent of days with a SIP order, based on the work of Berry et al. [7]. We categorized this measure of SIP policies to three levels (<30% of days, 30–59% of days, ≥60% of days) based on a combination of the histogram distribution and the sample size of each category to ensure that sample sizes were large enough within each cell.

#### 2.3.3. Covariates

Other variables included the respondent’s gender (male, female), age (65–84 years, 85 years and older), race (white non-Hispanic, non-white), living arrangement (alone, with others), Medicaid enrollment (yes, no), and marital status (separated or divorced versus widowed or never married) [22]. Never-married respondents were grouped with widowed respondents because the sample of those never married was too small to include separately. We used the combination of ADL and IADL impairment to reflect two levels of functional need: (1) least or moderately intense needs: no ADL needs and one or more IADL needs; and (2) most intense needs: any of the four ADL needs (eating, bathing, toileting, and dressing) [5,23]. We followed NHATS guidance to create a variable reflecting dementia classification that indicated no impairment versus possible or probable impairment [24].

### 2.4. Data Analyses

We computed weighted statistics using the weighting procedures recommended by NHATS guidelines [25]. We calculated weighted descriptive statistics for all variables and compared those characteristics by dementia status and activities of daily living needs. Then, we estimated a weighted multivariate multinomial logistic regression model with changes in caregiving as the outcome and SIP policies as the predictor, controlling for covariates. The reference outcome is no change (i.e., the same amount of support) during COVID-19. From the regression results, we calculated marginal probabilities and created a chart showing predictive margins of changes in caregiving. All analyses were conducted in Stata/MP version 17.0.

### 2.5. Sensitivity Analyses and Model Diagnostics

To assess the robustness of our findings, we conducted sensitivity analyses treating SIP exposure as both a continuous variable and a categorical variable (<30%, 30–74%, and ≥75% of days). Due to small cell sizes in some subgroups, analyses involving the ≥75% category could not be estimated. Results from the sensitivity analyses treating SIP exposure as a continuous variable were consistent with the primary findings (see Supplementary Table S1).

Multicollinearity was assessed using variance inflation factors (VIFs), and the independence of irrelevant alternatives (IIA) assumption was evaluated using the Hausman–McFadden test. Due to limitations of post-estimation diagnostics following survey-weighted multinomial logistic regression in Stata, IIA diagnostics were assessed using an equivalent multinomial logistic regression model without survey design adjustments. Overall model fit was assessed using design-adjusted Wald statistics from the survey-weighted multinomial logistic regression models. Model diagnostics indicated no evidence of problematic multicollinearity, poor model fit, or violation of the IIA assumption.

## 3. Results

### 3.1. Univariate Analyses

The weighted univariate results (Table 1) show that, compared to before COVID-19, more than half of unmarried respondents who required assistance (55.1%) reported no change in the help they received during the pandemic, whereas 36.7% reported receiving less help and 8.2% reported receiving more help. Changes in caregiving during COVID-19 compared to before did not differ by dementia status or by degree of support needs. However, a higher proportion of people without dementia reported receiving less help (39.3%) compared to those with possible or probable dementia (27.7%), whereas those with dementia were more likely to report no change (65.7%) compared to those without dementia (52.0%).

### 3.2. Multivariate Analyses

**Figure 2:**
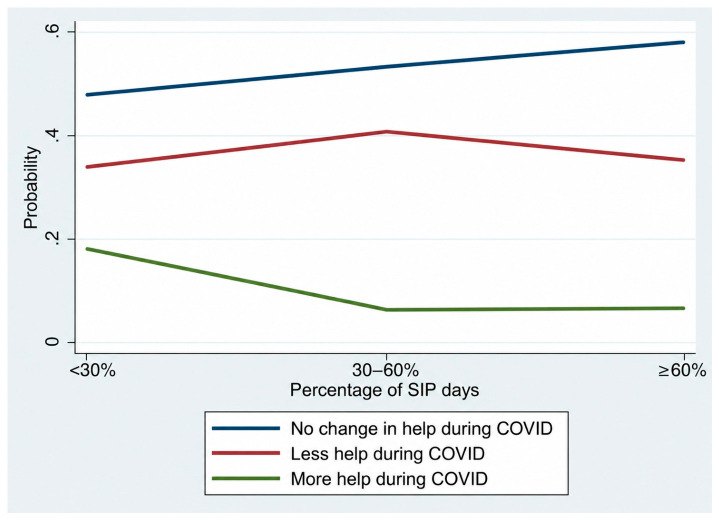
Predictive adjusted marginal probabilities of % of SIP days.

The weighted multinomial logistic regression results (Table 2) indicate that, compared to those living in areas with less than 30% SIP days, the adjusted marginal probability of receiving more help during COVID-19 was on average 12 percentage points lower for people living in areas with 30–59% SIP days (CI = [−0.22, −0.02]) and 12 percentage points lower for people with 60% or more SIP days (CI = [−0.22, −0.01]). Figure 2 depicts the adjusted marginal probabilities, demonstrating that the probability of receiving more help declined when the percentage of SIP days was more than 30%. The adjusted marginal probabilities for no change in help and for less help during COVID-19 were not statistically significant.

Only two covariates had statistically significant associations with changes in receiving help during COVID-19. The adjusted marginal probability of no change in help during COVID-19 was 12 percentage points higher for people who were widowed or never married compared to those who were separated or divorced (CI = [0.01, 0.24]). The adjusted marginal probability of receiving less help during COVID-19 was 15 percentage points higher for people living with others compared to those living alone (CI = [0.04, 0.26]).

## 4. Discussion

Based on the weighted analyses for our sample of 512 participants who had functional support needs and were not married from the Round 10 NHATS, we found that more than half reported no change in receiving help during COVID-19, one-third reported receiving less help during COVID-19, and over 8% reported receiving more help during COVID-19 than prior to the pandemic. People living in areas with a higher SIP percentage (30% or more) were less likely to experience an increase in help, compared to those living in areas with a lower SIP percentage (less than 30%). Moreover, people who were living with others were more likely to receive less help than people living alone.

Our study contributes to the mixed literature on caregiving changes during the COVID-19 pandemic. A comprehensive search of peer-reviewed studies found that caregivers of older adults did not feel fully supported by existing policies and recommendations during and immediately following the lockdown period of the COVID-19 pandemic [26], while another nationally representative study reported relatively little change in caregiving patterns during the pandemic [27]. A small survey conducted online with 109 people with disabilities, including younger adults, found that 54.0% of service changes were due to discontinuation, including loss of physical therapy, job coaching, community organizations, transportation, and peer supports. Other changes included a shift to virtual service delivery and family members taking the role of service providers [8]. This may help explain the potential mechanisms underlying the association between SIP policies and changes in caregiving. Similar to these previous studies, the data used in this study reflect the short-term impact of SIP policies, which may not capture longer-term adaptations in caregiving arrangements over the course of the pandemic.

Our results are also consistent with previous qualitative studies showing that some families chose to cease or reduce supportive care, expressed a desire to avoid future nursing home care where older adults could receive more caregiving support, and indicated hesitancy towards seeking home and community-based help until the pandemic was curbed [28]. Given the alarmingly high rates of infections and deaths in nursing homes, remaining in the community may have seemed like a safer option for older adults during this crisis, but doing so required substantial support for many of them [29]. Our results are similar to findings from a study using NHATS data that reported that 11.5% of respondents reported a decrease in the amount of care provided by family and friends [12]. Our population of people who were not married had a greater percentage who reported a decrease in support—36.7%—which suggests that this group’s support networks may have been more greatly impacted by COVID-19. One possible explanation could be that people who are not married rely more on non-family assistance, including paid caregivers [6,30]. The daily work of paid caregivers was disrupted or even halted during COVID-19 [16]. Another study of caregiving during COVID-19 found a decline in unpaid nonrelative helpers and no corresponding rise in paid helpers between 2019 and 2020 [10,31].

It is surprising to find that people living with others were more likely to receive less help compared to those living alone. Multigenerational households faced many challenges during COVID-19, including unstable employment or job loss, return of adult children to the household, concerns about within-household disease transmission, and interpersonal conflict [10,32]. Some family members may have limited their interactions to protect older adults, resulting in reduced caregiving support. In some households, school closures and increased childcare and education responsibilities may have reduced time available to support older adults in the household [33]. The adverse health, psychosocial, and financial impacts of COVID-19 on family caregivers may have also impacted the amount and quality of caregiving support they provided to family members with daily care needs [16,32]. In addition, evidence shows that neighborhood social relationships had heightened importance during the pandemic, and older adults relied on neighbors for practical and social support, particularly those living alone [34]. Together, these patterns may have led to older adults living with others perceiving they received less support, due to other demands and stressors in the household, while those living alone received more support, particularly from neighbors.

Our findings suggest that emergency preparedness and future SIP policies should prioritize maintaining continuity of home- and community-based caregiving services during public health crises to minimize disruptions in support for older adults living in the community. Targeted outreach and support may be particularly important for unmarried older adults and those who rely on nonfamily or paid caregivers, as these groups may be especially vulnerable to disruptions in caregiving during periods of restricted mobility and social distancing.

Strengths of this study include the robust NHATS methodology and innovatively linking NHATS geographic data with public SIP data at the state and county levels. However, this analysis is based on cross-sectional data and was not able to follow the trend of changes in SIP policies and their impact on caregiving in the long term. The causal inference cannot be ascertained in the study. Another limitation of this study was the use of complete-case analysis, as approximately 10% of observations were excluded due to missing data, which may have introduced selection bias if the missingness was not completely at random. Due to limitations of the small sample size, this study had to aggregate some variable categories (e.g., widowed and never married participants). Given that the study used an observational design and secondary data, we acknowledge that there may be residual confounding variables that were not fully captured in this study, such as socioeconomic status, geographic context, and health service availability. There is also the possibility of temporal misclassification or exposure heterogeneity across respondents interviewed at different time points, as the SIP exposure is defined as the percentage of days under county-level SIP orders between 24 February and 30 May 2020, while the NHATS Round 10 and COVID-19 supplemental data were collected across a broader time window extending into 2021. Because caregiving changes were assessed using self-reported changes in help across three broad categories, we were unable to determine the specific types, sources, or contexts of caregiving changes. Further qualitative and longitudinal research is needed to better understand the nature of these caregiving changes and the mechanisms through which SIP policies may have influenced caregiving patterns.

## 5. Conclusions

It is important to address the caregiving needs of older adults with disabilities during public health emergencies and to examine how disruptions caused by policies such as SIP orders were related to the provision of help for people who rely on family and friends for support. Disruptions caused by public health emergencies can negatively impact support systems, including community-based programs, healthcare services, and the availability of friends and family. A network of family, unpaid non-family, and paid support is necessary to ensure care needs are met, especially during public health emergencies. Policymakers, community leaders, and healthcare providers need to develop resilient programs and plans to ensure that the people they serve have adequate support, especially during significant disruptions.

## Figures and Tables

**Table 1:** Weighted characteristics of NHATS respondents with long-term services and support needs who were not married: (1) full sample: (2) by dementia status; (3) by ADL/IADL, 2020–2021.

		By Dementia Status		By ADL/IADL	
	Full Sample	No Dementia	Possible/Probable Dementia	Least/Moderate Needs	Most Intense Needs
	(Unweighted n = 512)	(Unweighted n = 356)	(Unweighted n = 156)	(Unweighted n = 390)	(Unweighted n = 122)
Change in caregiving: During COVID-19 vs. Before					
no change in help during COVID	55.1	52.0	65.7	54.4	58.8
less help during COVID	36.7	39.3	27.7	37.7	31.4
more help during COVID	8.2	8.7	6.6	7.9	9.9
Percentage of “Shelter-in-Place” Policy Days					
<30%, %	16.6	14.9	22.7	14.1	20.5
30–59%, %	18.8	30.9	21.6	30.3	21.5
≥60%, %	54.5	54.2	55.7	55.6	49.1
Dementia Status					
No Impairment, %	77.4	NA	NA	86.5	32.3
Possible or probable, %	22.6	NA	NA	13.5	67.7
ADL/IADL Needs					
Least and moderate intense needs, %	83.3	93.0	49.8	NA	NA
Most intense needs, %	16.8	7.0	50.3	NA	NA
Gender					
Male, %	24.4	23.2	28.5	25.0	21.5
Female, %	75.6	76.8	71.5	75.0	78.5
Age					
65 to 84 years, %	66.5	74.8	38.2	71.2	43.3
85+ years, %	33.5	25.3	61.8	28.8	56.7
Race					
White, non-Hispanic, %	80.3	83.4	69.6	83.2	65.8
Non-white %	19.7	16.6	30.4	16.8	34.2
Marital Status					
Separated or divorced, %	28.3	30.9	19.3	29.5	22.5
Widowed or never married, %	71.7	69.1	80.7	70.6	77.5
Living Arrangement					
Alone, %	55.0	57.9	45.0	60.2	29.0
With others, %	45.0	42.2	55.0	39.8	71.0
Enrolled in Medicaid					
Yes, %	11.7	7.3	26.8	6.6	37.1
No, %	88.3	73.2	92.7	93.4	62.9

Note: NA means not applicable.

**Table 2:** Weighted multinomial logistic regression for factors related to changes in caregiving during COVID-19 among NHATS respondents with long-term services and support needs who were not married, 2020–2021.

	No Change/Same During COVID-19 (Unweighted N = 282)		Less Help During COVID-19 (Unweighted N = 182)		More Help During COVID-19 (Unweighted N = 48)	
	Adjusted Marginal Probability	95% Confidence Interval	Adjusted Marginal Probability	95% Confidence Interval	Adjusted Marginal Probability	95% Confidence Interval
Percentage of “Shelter-in-Place” policy days (ref ≤ 30%)						
30–59%	0.05	(−0.10, 0.21)	0.07	(−0.08, 0.21)	−0.12	(−0.22, −0.02)
≥60%	0.10	(−0.04, 0.25)	0.01	(−0.12, 0.14)	−0.12	(−0.22, −0.01)
Dementia Status (ref = No dementia)						
Possible or probable dementia	0.12	(−0.01, 0.25)	−0.07	(−0.21, 0.07)	−0.05	(−0.12, 0.01)
ADL/IADL Needs (ref = Least or moderate intense needs)						
Most intense needs	0.01	(−0.14, 0.15)	−0.04	(−0.21, 0.13)	0.03	(−0.09, 0.15)
Gender (ref = Male)						
Female	−0.05	(−0.21, 0.10)	0.08	(−0.05, 0.22)	−0.03	(−0.09, 0.03)
Age (ref = 65 to 84 years)						
85+ years	0.04	(−0.08, 0.14)	−0.09	(−0.18, 0.00)	−0.05	(−0.02, 0.13)
Race (ref = White, non-Hispanic)						
Non-white	0.04	(−0.09, 0.17)	−0.06	(−0.17, 0.05)	0.02	(−0.05, 0.09)
Marital Status (ref = Separated or Divorced)						
Widowed or never married	0.12	(0.01, 0.24)	−0.09	(−0.20, 0.02)	−0.03	(−0.11, 0.05)
Living Arrangement (ref = Alone)						
With others	−0.11	(−0.22, 0.01)	0.15	(0.04, 0.26)	−0.04	(−0.10, 0.02)
Enrolled in Medicaid (ref = No)						
Yes	−0.03	(−0.21, 0.16)	0.03	(−0.16, 0.22)	0.00	(−0.07, 0.07)

## Data Availability

Study data are not publicly available because the National Health and Aging Trends Study (NHATS) restricted data requires specific procedures for access due to confidentiality and privacy concerns. Access to these restricted files is granted to approved researchers through a partnership with the Michigan Center on the Demography of Aging (MiCDA) at the University of Michigan.

## Supplementary Materials

The following supporting information can be downloaded at: https://www.mdpi.com/article/10.3390/ijerph23070825/s1, Table S1: Weighted multinomial logistic regression for factors (using continuous percentage of “Shelter-in-Place” policies days) related to changes in caregiving during COVID-19 among NHATS respondents with long-term services and support needs who were not married, 2020–2021.

## Abbreviations

The following abbreviations are used in this manuscript:

SIP	Shelter-in-Place
NHATS	National Health and Aging Trends Survey
ADLs	Activities of daily living
IADLs	Instrumental activities of daily living
