# Supplementary material for: “Shelter-in-Place” Policies and Changes in Caregiving for Older Adults During the COVID-19 Pandemic
Source: Int J Environ Res Public Health. 2026 Jun 23;23(7):825. doi: 10.3390/ijerph23070825 (PMC13410357; doi:10.3390/ijerph23070825)
Supplement: Supplementary file 1 [file ijerph-23-00825-s001.zip › ijerph-4248339-supplementary.pdf]

Table S1. Weighted multinomial logistic regression for factors (using continuous percentage of "Shelter-in-Place" policies days) related to changes in caregiving during COVID-19 among NHATS respondents with long-term services and support needs who were not married, 2020-2021.

	No Change/Same during COVID-19 (Unweighted N=282)		Less help during COVID-19 (Unweighted N=182)		More help during COVID-19 (Unweighted N=48)	
	Adjusted Marginal Probability	95% Confidence Interval	Adjusted Marginal Probability	95% Confidence Interval	Adjusted Marginal Probability	95% Confidence Interval
**Percentage of "Shelter-in-Place" policies days (continuous)**	0.002	(-0.000,0.004)	-0.001	(-0.003, 0.002)	-0.001	(-0.002, 0.000)
**Dementia Status (ref = No dementia)**						
Possible or probable dementia	0.12	(-0.01, 0.25)	-0.07	(-0.21, 0.07)	-0.05	(-0.12, 0.02)
**ADL/IADL Needs (ref = Least or moderate intense needs)**						
Most intense needs	0.01	(-0.14, 0.15)	-0.05	(-0.21, 0.07)	0.05	(-0.10, 0.19)
**Gender (ref = Male)**						
Female	-0.05	(-0.20, 0.10)	0.08	(-0.06, 0.21)	-0.02	(-0.09, 0.04)
**Age (ref = 65 to 84 years)**						
85+ years	0.04	(-0.07, 0.15)	-0.09	(-0.18, 0.00)	0.05	(-0.02, 0.12)
**Race (ref = White, non-Hispanic)**						
Non-white	0.04	(-0.09, 0.17)	-0.07	(-0.18, 0.00)	0.02	(-0.05, 0.09)
**Marital Status (ref = Separated or Divorced)**						
Widowed or never married	0.13	(0.01, 0.24)	-0.09	(-0.21, 0.03)	-0.04	(-0.12, 0.05)
**Living Arrangement (ref = Alone)**						
With others	-0.11	(-0.22, 0.01)	0.15	(0.04, 0.26)	-0.04	(-0.10, 0.01)
**Enrolled in Medicaid (ref = No)**						
Yes	-0.03	(-0.21, 0.15)	0.03	(-0.16, 0.22)	-0.00	(-0.07, 0.07)
